# The Effect of Chain Structures on the Crystallization Behavior and Membrane Formation of Poly(Vinylidene Fluoride) Copolymers

**DOI:** 10.3390/membranes4020243

**Published:** 2014-05-19

**Authors:** Wenzhong Ma, Haoge Yuan, Xiaolin Wang

**Affiliations:** 1 Beijing Key Laboratory of Membrane Materials and Engineering, Department of Chemical Engineering, Tsinghua University, 1 Tsinghuayuan, Beijing 100084, China; E-Mail: yhg12@mails.tsinghua.edu.cn; 2 College of Materials Science and Engineering, Changzhou University, 1 Gehu Road, Changzhou 213164, China

**Keywords:** poly(vinylidene fluoride) copolymers, membrane, thermally-induced phase separation

## Abstract

The crystallization behaviors of two copolymers of PVDF were studied, and the effect of copolymerized chains on the crystallization behavior was investigated. The results indicated that both copolymers had a lowered crystallization temperature and crystallinity. The crystallization rate was improved by the copolymer with symmetrical units in PVDF chains, but hindered by asymmetrical units, compared with the neat PVDF. The symmetrical units in PVDF chains favored the β-crystals with fiber-like structures. According to the solubility parameter rule, methyl salicylate (MS) can be chosen as a diluent for PVDF copolymers. Both diluted systems had liquid-liquid (L-L) regions in the phase diagrams, which was due to the lowered crystallization temperature.

## 1. Introduction

Poly(vinylidene fluoride) (PVDF), a semi-crystalline polymer with excellent physical and chemical properties and a good thermal stability, has been used in many applications [1]. Since the PVDF molecular chain is composed of carbon backbones with both sides of the hydrogen atom and fluorine atom symmetrically arranged, PVDF is easy to crystallize. At least four known crystalline phases of PVDF, α, β, γ and δ, can be obtained, depending on the competition between the phase separation and the crystallization process [2] and the solvent evaporation rate when PVDF is crystallized from a solution [3,4]. This implies that when phase separation occurs, the kinetic parameters (including quenching temperatures, the time limit for each phase separation region) will play an important role in the ultimate membrane structure. According to different polymerization methods, PVDF usually has regular head-to-tail sequences, but also has a reversed monomeric addition, leading to head-to-head and tail-to-tail defects [5]. Lovinger *et al.* reported that such head-to-head or tail-to-tail defects will affect the crystalline form of PVDF [6]. Thus, the molecular chain structure would significantly influence the crystallization.

Nowadays, thermally-induced phase separation (TIPS), as one of the common techniques for membrane preparation, is widely used, because of the advantages over the conventional membrane preparation technique [7,8]. During the TIPS process, the liquid-liquid (L-L) phase separation, solid-liquid (S-L) phase separation or a combination of both of these two separations, followed by polymer crystallization, offers great flexibility in controlling the microscopic morphology of polymeric membranes [7,8,9,10,11]. In the membrane preparation by TIPS, selecting a proper solvent is one of the key factors for controlling the pore structure. In previous work [12,13], a wide L-L phase separation can be obtained in PVDF/diphenyl ketone (DPK) and diphenyl carbonate (DPC) systems. Most diluted systems undergo an S-L phase separation associated with the crystallization of PVDF, which is due to the strong interactions between the PVDF and diluent molecules. Establishing these relationships requires a better understanding of how cooperative interactions between the crystallization and the phase separation mechanism can lead to interconnected networks.

The main aim of this work is to investigate the effect of the polymer chain structure on the mechanism of the crystallization and membrane structure via the TIPS process. Herein, methyl salicylate (MS) was used as a diluent, which is nonsolvent at room temperature, but a good solvent athigh temperatures.

## 2. Results and Discussion

**Figure 1:**
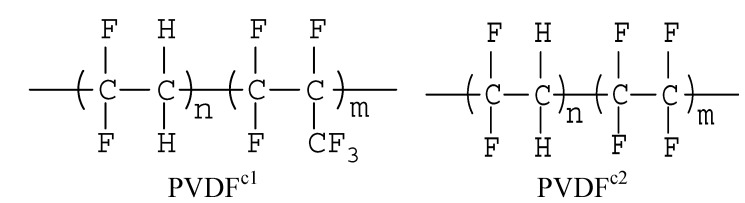
Molecular structures of the PVDF copolymers, poly(vinylidene fluoride-*co*-hexafluoropropylene) [PVDF-HFP (PVDF^c1^)] and poly(vinylidene fluoride-*co*-tetrafluoroethylene) [P(VDF-TFE) (PVDF^c2^)].

In this work, two kinds of PVDF copolymer structures were used (as shown in Figure 1). PVDF^c1^ and PVDF^c2^ are the abbreviation of poly(vinylidene fluoride-*co*-hexafluoropropylene) (PVDF-HFP) and poly(vinylidene fluoride-*co*-tetrafluoroethylene) [P(VDF-TFE)], respectively. Compared with the PVDF homopolymer structure, an asymmetrical unit of fluoropropylene (HFP) was incorporated into the main constituent vinylidene fluoride (VDF) blocks in PVDF^c1^, but a symmetrical unit of tetrafluoroethylene (TFE) in PVDF^c2^. Thus, the regularity of the copolymer chain is totally different from the homo-PVDF. In the following discussion, the crystallization behaviors of copolymers will be investigated.

### 2.1. The Effect of Chain Structures on the Crystallization Behavior of PVDF Copolymers

DSC results, including the crystallization of homo-PVDF and co-PVDF at a cooling rate of 2.5 °C/min when cooling from 200 °C and subsequent melting at a heating rate of 10 °C/min, are summarized in Table 1.

**Table 1:** DSC crystallization results of homer- and co-PVDF crystallizing from the melt at a cooling rate of 2.5 °C/min.

Sample	*T_c_^on^* (°C)	*T_c_^p^* (°C)	*T_c_^f^* (°C)	Δ*T_c_* (°C)	*T_m_^p^* (°C) *	Δ*H_m_* (J·g^−1^)	*X_c_* (%)
PVDF	141.3	138.1	135.8	3.2	174.2	49.8	47.7
PVDF^c1^	133.9	129.2	126.0	4.7	162.8	29.2	27.9
PVDF^c2^	117.5	116.2	113.8	1.3	141.0	37.8	37.2

*T_c_^on^*, onset crystallization temperature; *T_c_^p^*, peak crystallization temperature; *T_c_^f^*, final crystallization temperature; Δ*T_c_* = *T_c_^on^* − *T_c_^p^*; Δ*H_m_*, melting enthalpy; *X_c_*, crystallinity; * results obtained under a scanning rate of 10 °C/min.

As illustrated in Table 1, the crystallization temperatures of those two copolymers (including the values of *T_c_^on^*, *T_c_^p^* and *T_c_^f^*) are lower than homo-PVDF. This can be attributed to the lower degree of structural regularity for co-PVDF. The crystallinity of each co-PVDF is lower than the homo-PVDF, which indicates that the comonomer unit introduced into the PVDF chain retards the polymer chains crystallizing into lamellae. The difference between the onset and peak crystallization temperature, ∆*T_c_*, of PVDF^c1^ is 4.7 °C, which is higher than PVDF^c2^ and also higher than homo-PVDF. The value, ∆*T_c_*, of PVDF^c2^ is 1.3 °C, which is much lower than homo-PVDF. The lower the value, ∆*T_c_*, is, the faster the crystallization rate is [14]. Thus, the crystallization rate is in the order of PVDF^c2^ > homo-PVDF > PVDF^c1^. This can be explained by the different crystallization mechanisms, which will be discussed in the following section.

**Figure 2:**
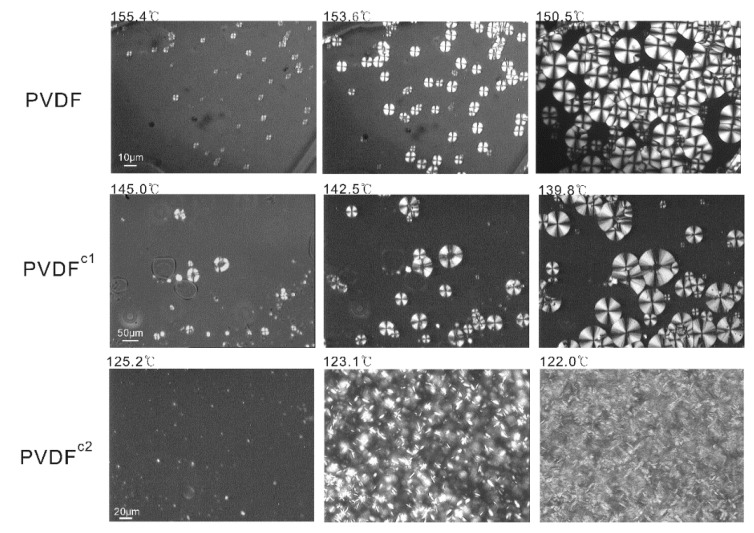
Polarized optical micrograph showing the crystalline morphology of homer- and co-PVDF crystallizing from the melt at a cooling rate of 0.5 °C/min.

Figure 2 shows the typical spherulitic texture of homo-PVDF and PVDF^c1^ crystallizing from the melt at a cooling rate of 0.5 °C/min. When cooling from the melt, both homo-PVDF and PVDF^c1^ reveal well-defined nucleation and growth processes. The spherulites of these two samples impinge each other in about 10 min after crystallization begins. However, as for PVDF^c2^, ribbon-like structures that mostly arrange into concentric needles are observed, and the growth of these crystals nearly completes in 6 min. This result indicates that the HFP unit in the PVDF chain did not change the crystallization style, which is three-dimensional crystallites (spherulitic structure). The reduced crystallization temperature, crystallinity and crystallization rate can be assigned to the asymmetry of the HFP unit in the chain. On the contrary, the TFE unit in the PVDF chain changed the crystallization style from three-dimensional crystallites to one-dimensional crystallites (rod-like or needle-like crystals). This can be attributed to the fast crystallization rate of the TEF chains, which is similar to the crystallization of PTFE [15]. Therefore, the crystallization rate of PVDF^c2^ is faster than PVDF^c1^ and homo-PVDF, which agrees with the DSC results.

**Figure 3:**
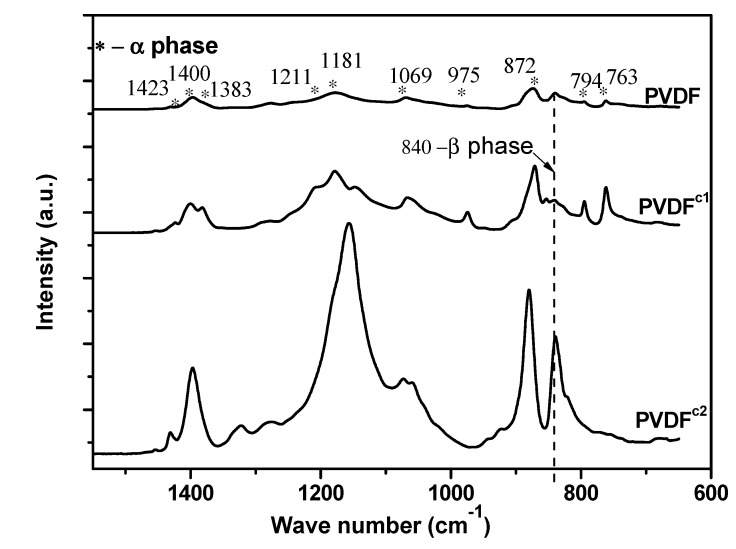
FTIR spectra of homer- and co-PVDF samples crystallizing from the melt.

In this study, the crystalline phases of PVDF in the diluted samples are identified by FTIR spectroscopy (Figure 3). Homo-PVDF and PVDF^c1^ have well-defined absorption bands at 1423, 1400, 1383, 1211, 1181, 1069, 975, 872, 794 and 763 cm^−1^. As reported, these IR absorption bands represent the characteristic spectrum of the α phase of the PVDF crystal [16,17]. This indicates that only the crystallization of α phase PVDF predominates in the crystallization in PVDF^c1^. However, with regard to PVDF^c2^, except for the absorption bands of the α phase PVDF, an additional strong absorption band at 840 cm^−1^ was observed, which is characteristic of the β phase of PVDF. Therefore, the TFE unit in the PVDF chain favors the β-PVDF crystal formation, which confirms the polarized optical micrograph (POM) observation that the needle-like crystals are the β-PVDF.

**Figure 4:**
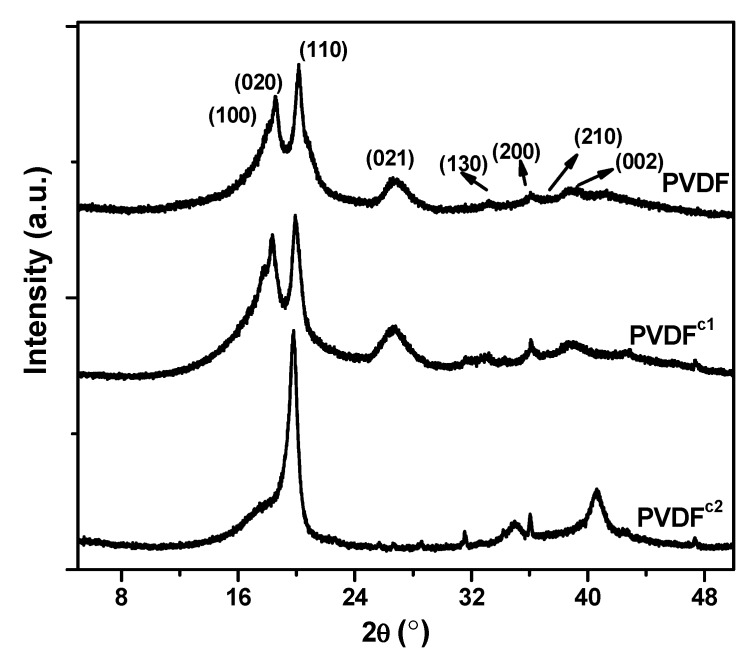
X-ray diffractograms of homer- and co-PVDF samples crystallizing from the melt.

To confirm the crystalline phase in the co-PVDF samples, X-ray diffraction was carried out (Figure 4). According to the researchers’ work [16,18], the peaks at 2θ = 17.75°, 18.36°, 19.96°, 26.58°, 33.10°, 36.99°, 38.64° and 39.00° in the curve for the homo-PVDF sample represent the diffractions in the planes, (100), (020), (110), (021), (130), (200), (210) and (002), respectively, which are all characteristic of the α phase of PVDF. For the PVDF^c1^ sample, the diffraction peaks are the same as the homo-PVDF. However, for the PVDF^c2^ sample, only plane (110) has a strong diffraction peak, and a small peak at around 40° is also different from the other two samples, which confirms only α phase crystals in the PVDF^c1^, but mainly β phase crystals in the PVDF^c1^ [19].

In the TIPS process, the diluent is crucial in determining the polymer crystallization process and the resulting membrane morphology. Table 2 shows the solubility parameters for homo-PVDF, co-PVDF and MS. As shown in Table 2, the difference in the solubility parameters between the PVDF and MS is little, so MS can be selected as the diluent. However, an obvious spherulitic structure was obtained in the membrane, which is due to the strong interaction between the MS molecule and the PVDF chain [20]. As calculated by molecular dynamics simulations, the solubility parameters for PVDF^c1^ and PVDF^c2^ are 13.5 and 15.1 MPa^1/2^, respectively [11]. This indicates weak interactions between the polymer chain and MS molecules, which is due to the presence of the copolymerized units.

**Table 2:** Solubility parameters. MS, methyl salicylate.

Materials	Solubility parameter δ (MPa^1/2^) [11]
PVDF	19.2
PVDF^c1^	13.5 *
PVDF^c2^	15.1 *
MS	21.7

* calculated by molecular dynamics simulations.

### 2.2. The Effect of Chain Structures on the Membrane Formation of PVDF Copolymers

**Figure 5:**
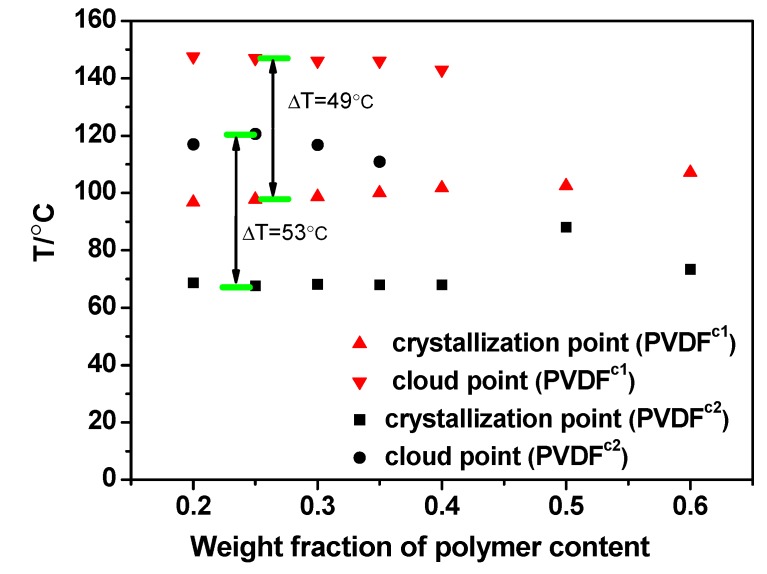
Phase diagram for PVDF copolymer/MS systems.

The values of dynamic crystallization temperature and the cloud point are plotted to obtain the phase diagrams of the co-PVDF/MS system, as shown in Figure 5. The phase diagram of thehomo-PVDF/MS system has been reported in previous work [10]. Although the L-L region can be obtained by adding PMMA, which lowers the crystallization temperature of PVDF, the crystallization of PVDF in the MS dilutions is still obvious, because of the strong interaction between the PVDF chains and MS molecules.

Due to the weak polymer-diluent interaction, the phase diagram of the co-PVDF/MS-diluted system shows the upper critical solution temperature (UCST) type L-L phase behavior [7], as shown in Figure 5. Compared with a PVDF/MS system [10], an obvious wide L-L phase separation region is observed, in which the monotectic point, φ_m_ (the intersection of the crystallization temperature curve with the cloud curve) [21], of PVDF^c1^ and PVDF^c2^ is around 50 wt% and 40 wt%, respectively. For the PVDF^c2^/MS system, the cloud points are about 20 °C lower than the PVDF^c1^/MS system, which is due to a weaker interaction between the PVDF^c1^ and MS molecules, indicated by the aforementioned solubility analysis. Similarly, the crystallization temperature of PVDF^c1^ is higher than PVDF^c2^. This can be attributed to the crystalline form changing in the presence of the TEF unit in the PVDF chains. For both systems, for samples with a lower polymer concentration (<φ_m_), the dilution effect induces the L-L phase separation with a horizontal crystallization curve, but in a high polymer concentration (>φ_m_) region, the L-L phase separation will be arrested by the crystallization.

**Figure 6:**
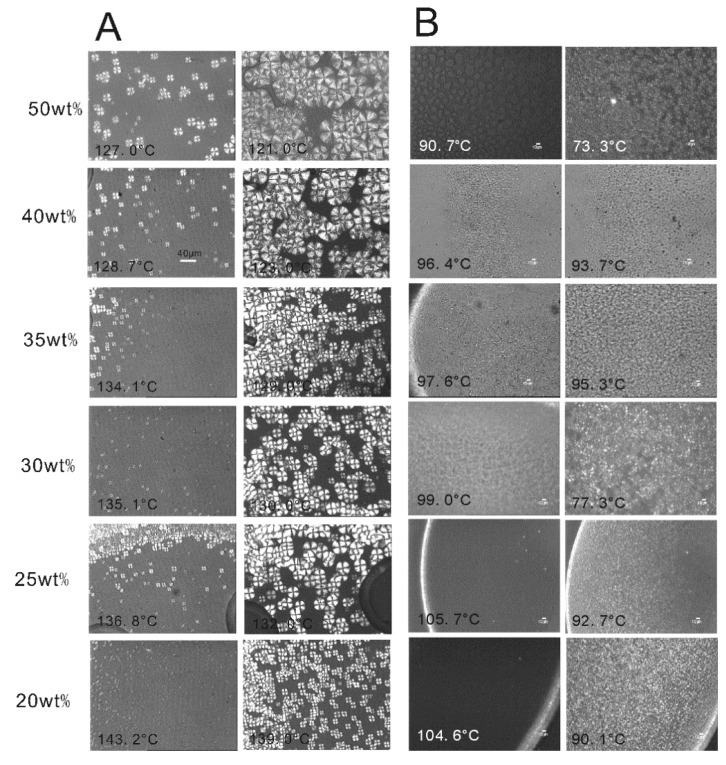
Polarized optical micrographs showing the spherulitic morphology of PVDF copolymer/MS dilutions, crystallized from the melt at a cooling rate of 1 °C/min: (**A**) PVDF^c1^/MS dilution; (**B**) PVDF^c2^/MS dilution.

As shown in Figure 6, the morphologies of PVDF copolymer/MS dilutions crystallizing from the melt at a cooling rate of 1 °C/min were observed by polarized light microscopy. This reveals the well-defined large spherulites of PVDF^c1^ in MS, which is the same as is observed in Figure 2. In the MS dilution, the crystal size is smaller than in the pure polymer sample (Figure 2), which is due to the supercooling in the ice water bath for MS dilution samples. For the PVDF^c1^/MS-diluted system, the number of nuclei and the impinged spherulitic crystal size is decreased with an increase of the polymer concentration, because the entangled chains and highly viscous state restrain chain mobility [22].

With regard to the PVDF^c2^/MS-diluted system, the number of crystal nuclei is decreased with an increase of the polymer concentration, which is similar to the PVDF^c1^/MS dilution, but the crystal size is independent of the polymer concentration, because of the ribbon-like structures. Similar to the neat PVDF^c2^ sample observation (Figure 2), this ribbon-like crystal cannot grow into a larger size. However, the number of crystals is decreased with an increase of the polymer concentration, which still can be explained by the entangled chains and highly viscous state restraining chain mobility in a high polymer condition.

**Figure 7:**
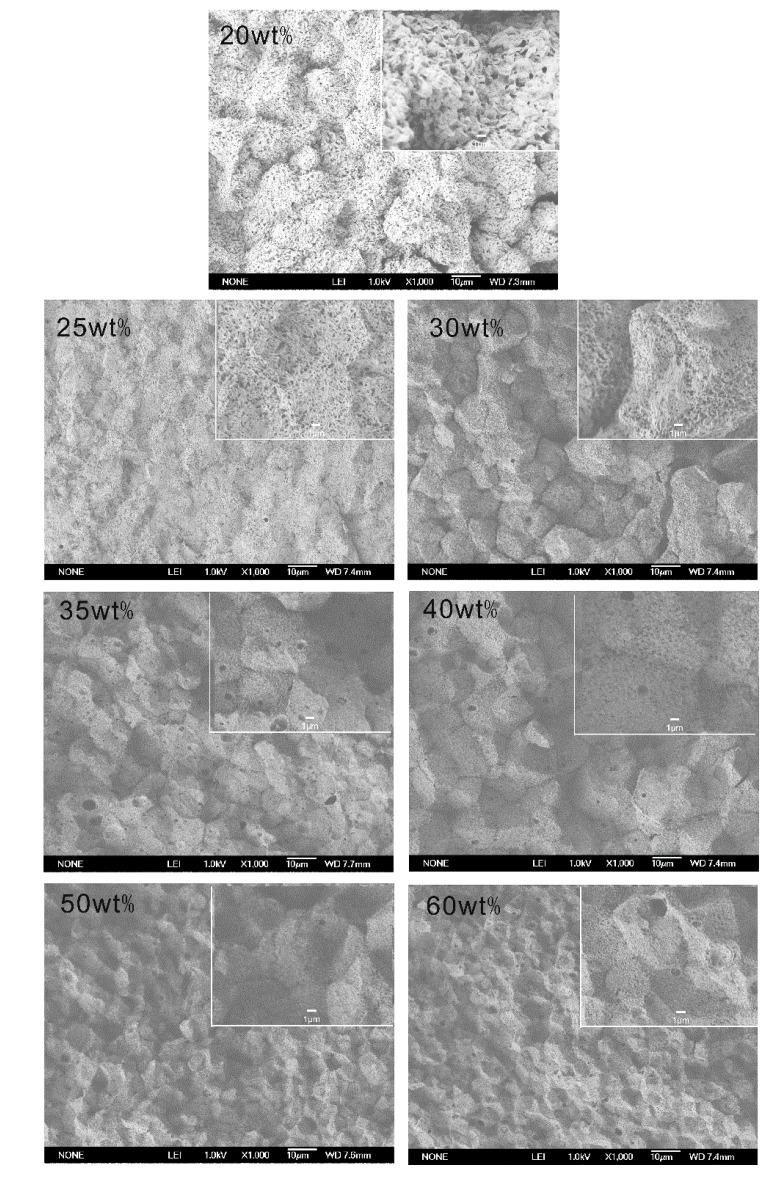
SEM micrographs of PVDF^c1^ membranes prepared from MS dilutions quenched in the ice water bath (the polymer content is marked in the upper left corner of each picture).

**Figure 8:**
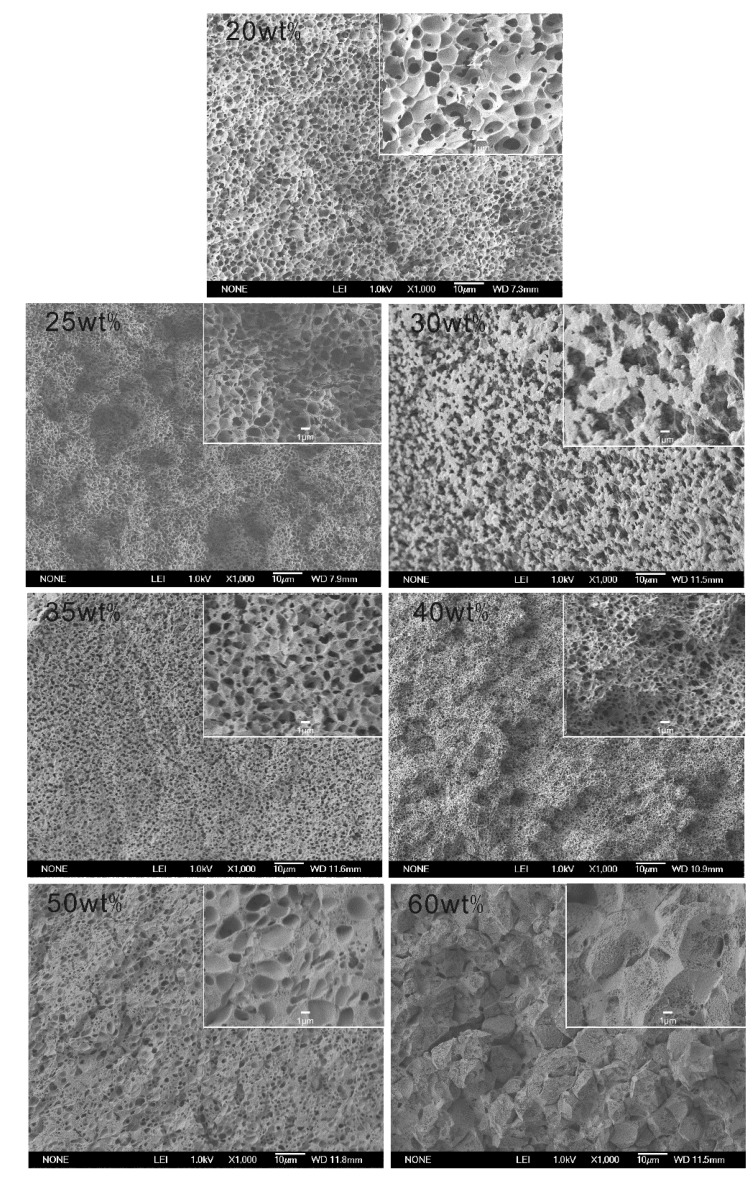
SEM micrographs of PVDF^c2^ membranes prepared from MS dilutions quenched in the ice water bath (the polymer content is marked in the upper left corner of each picture).

The membrane structures of PVDF^c1^ and PVDF^c2^ are shown in Figure 7 and Figure 8, respectively. The membranes obtained from these two systems have a micropore structure in the cross-section.

As for the PVDF^c1^ membranes, due to the growth of spherulites, the membrane structure is similar to the homo-PVDF membrane [10], which is full of spherulites with small pores in it (Figure 7). For the PVDF^c1^/MS dilution, the largest temperature ranging from the cloud point to the crystallization point (∆*T*) is 49 °C, which is smaller than the PVDF^c1^/MS dilution (∆*T* = 53 °C). This indicates that the region of the L-L phase separation is still small (Figure 5). Therefore, the L-L phase separation will be quickly arrested by the crystallization of PVDF^c1^, leading to the rejection of the liquid diluent to inter- and intra-spherulitic regions. When the polymer concentration is lower than 40 wt%, the small pores formed through the L-L phase separation are observed, but a dense and small spherulitic structure is obtained when the polymer concentration is higher than 40 wt%. Thus, the polymer concentration (40 wt%), which shows that the dramatic morphology change is lower than the monotectic point, φ_m_. This can be attributed to the high degree supercooling (quenching in the ice water) condition. When the PVDF^c1^ concentration is higher than 40 wt%, a smaller pore size and spherulite size are observed, because of the limited mobility of polymer chains.

With regard to the PVDF^c2^/MS system, micropore structures derived from an L-L phase separation are more obvious than the PVDF^c1^/MS system. As shown in Figure 8, at a polymer concentration of 35 wt%, the pore morphology observed microscopically changed dramatically, indicating the L-L phase separation before the crystallization and that the monotectic point, φ_m_, is around 35 wt%. However, this is lower than that determined by the phase diagram (Figure 5). This also can be attributed to the high degree supercooling (quenching in the ice water) condition. When the PVDF^c2^ concentration is higher than 35 wt%, the crystallization occurs before the L-L phase separation. In this circumstance, the S-L phase separation could be approximately considered. However, the size of the spherulitic structure (the same as the α-crystal morphology) is increased with the polymer concentration, which is different from the PVDF^c1^ membrane. This can be explained by the crystal formation mechanism of PVDF^c2^. As discussed in the crystallization behavior of PVDF^c2^, mainly β-crystals are formed, but still, small α-crystal exists. Therefore, when the PVDF^c2^ concentration is higher, the spherulitic structure becomes more obvious.

**Figure 9:**
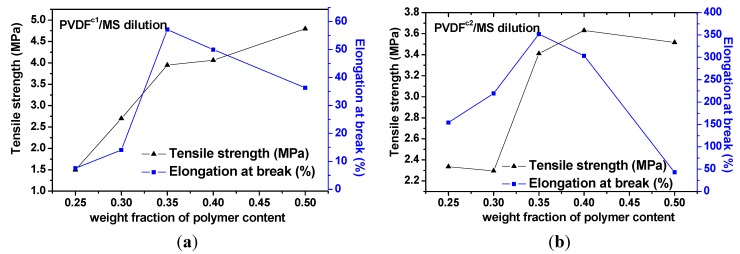
Tensile strength and elongation at break for membranes derived from PVDF copolymer/MS dilutions with various polymer contents that were quenched, from the melt to the ice water bath. (**a**) PVDF^c1^/MS diluted system; (**b**) PVDF^c2^/MS diluted system.

Figure 9 shows the tensile strength and elongation at break of PVDF^c1^ and PVDF^c2^ membranes obtained from various polymer concentrations by quenching in the ice water bath. The tensile strength for PVDF^c1^ and PVDF^c2^ membranes are increased with the polymer concentration increasing. The highest elongation at break of PVDF^c1^ and PVDF^c2^ membranes with a polymer concentration of 35 wt% is 55% and 350%, respectively. The enhanced performances of elongation at break of PVDF^c2^ can be attributed to the small and ribbon-like crystals induced by the TFE unit in chains. For the elongation at break of PVDF^c1^, it is nearly the same as the homo-PVDF membrane [23], which confirms that the spherulitic structure in the membrane can reduce the elongation at break.

## 3. Experimental Section

### 3.1. Materials

PVDF (Kynar K-761), in powder form, was supplied by Elf Atochem of North America Inc. (Calvert, KY, USA). PVDF copolymers: PVDF^c1^ (Solvay 11008) pallets were supplied by Solvay (Shanghai, China) company, and PVDF^c2^ pallets were supplied by Daikin (Beijing, China) company. The structures of these two copolymers are shown in Figure 1. Methyl salicylate (MS) was purchased from Sinopharm Chemical Reagent Co. Ltd. (Shanghai, China).

### 3.2. Sample Preparations

PVDF copolymer/MS dilutions with various mass ratios were kept in 20 mL standard ampoules with an argon atmosphere condition. The ampoules were sealed and kept in an oven at 200 °C for 48 h to make homogeneous solutions Then, the ampoules were quenched in liquid nitrogen to get PVDF copolymer/MS mixtures. After an extraction of MS from those mixtures through ethanol immersion, the ultimate microporous membranes were obtained.

### 3.3. Characterization Techniques

Fourier transform infrared spectroscopy (FTIR) spectra were obtained by using a Nicolet 6700 with 4 cm^−1^ resolution, 64 scans. FTIR-attenuated total reflection (ATR) spectra were applied in this work. Before this measurement, a thin polymer film with an average thickness of 0.5 mm was prepared by compression molding at 200 °C and quickly solidifying in cold water (25 °C).

Wide-angle X-ray diffraction (WAXD) was done in a Bruker D8-Advance diffractometer (Cu K_α_ radiation, 40 kV and 40 mA). The scanning angle ranged from 5° to 50° with a scanning velocity of 4°/min. The sample preparation is the same as the FTIR measurement.

Differential scanning calorimetry (DSC) analyses were made on a TA Instruments Q-200 differential scanning calorimeter in a dry nitrogen atmosphere. For the sample measurement, an appropriate amount of sample was sealed into an aluminum pan. Before the melting tests, the thermal history was erased by a quick heating to 200 °C. Then, the crystallization curve was obtained at a cooling rate of 2.5 °C/min to 40 °C. The onset of the exothermic peak during the cooling was taken as the dynamic crystallization temperature. After maintaining at 40 °C for 2 min, the mixtures were heated up to 200 °C at a rate of 10 °C/min. The crystallinity of PVDF (X_c_) was calculated as in previous work [23].

The morphology development of spherulites, which appears as bright areas under polarized light in the dark background of PVDF copolymer and MS diluted mixtures, was observed under cross-polarizers using a polarizing microscope (Olympus BX 51) equipped with a temperature controller (Linkam THMS 600). An appropriate amount of the sample was inserted between two microscope slides (with diameter of 20 mm), melted up to 200 °C and kept at this temperature for 5 min to erase the thermal history of the sample. Then, it was cooled down to 50 °C at a slow rate of 0.5–1.0 °C/min. Photomicrographs of growing spherulites were taken when the crystallization completed. The melt crystallization of the sample was carried out on the Linkam hot stage. Images of spherulitic growth during the crystallization were obtained by the polarizing microscope, and the diameter of the developed spherulite was measured with time using a video recording system (Linksys 32, Linkam Scientific Instruments, Ltd.). The cloud point curve is usually determined as follows. The homogeneous polymer blend/diluent sample was chopped into small pieces and placed between a pair of microscope cover slips. The sample was heated to 180 °C for 10 min to assure homogeneity. Then, it was cooled to the solidification state at a manual control rate of 1 °C/min. Cloud points were determined visually by noting the appearance of turbidity under the microscope.

Membranes were fractured in liquid nitrogen, and the surfaces were sputtered with Au in vacuum. Then, the SEM micrographs of dried and gold-coated cross-sections were taken with a JEOL JSM-7401 instrument.

The tensile strength of the membrane was measured by a universal testing machine (Shimadzu AGS-100A) equipped with a 5 kg load cell. Before the tests, the membranes with 0.2 mm thickness were cut into 10 × 3 mm^2^ strips. The crosshead speed was controlled at 2 mm/min. The average value of the tensile strength was calculated by measuring three samples for each batch of the membranes.

## 4. Conclusions

The effect of chain structure and membrane formation via the TIPS method was investigated in this work. The results are listed as follows:

(1) The main molecular chain structure was changed by the HFP and TFE unit. The crystallization rate was improved by the copolymer with symmetrical units in PVDF chains, but hindered by asymmetrical units, compared with the neat PVDF. The symmetrical units in PVDF chains favored the β-crystals with fiber-like structures.
(2) The interaction between PVDF copolymer and MS was weaker than between homo-PVDF and MS, so that a wide L-L phase separation was observed. The phase diagram of co-PVDF/MS revealed that the monotectic point, φ_m_, of PVDF^c1^ and PVDF^c2^ is around 50 wt% and 40 wt%, respectively. For the PVDF^c1^ membrane, mainly due to the presence of α-spherulites, the cross-section of the membrane was full of spherulites with small pores in it; while no significant spherulitic structures appeared in the PVDF^c2^ membrane, which is conducive to the form of the bicontinuous pore membrane.
(3) The tensile strength for PVDF^c1^ and PVDF^c2^ membranes was increased with the polymer concentration increasing. The TFE unit in chains favored the β-PVDF crystals, which enhanced the performances of elongation at break of PVDF^c2^, but for the PVDF^c1^ membrane, the HFP unit maintained α-PVDF crystals, with no improvement of the elongation at break, compared with the homo-PVDF membrane.

## References

[1] Seiler D.A., Scheirs J. (1997). PVDF in the chemical process industry. Modern Fluoropolymers.

[2] Van de Witte P., Dijkstra P.J., van den Berg J.W.A., Feijen J. (1996). Phase separation processes in polymer solutions in relation to membrane formation. J. Membr. Sci..

[3] Magalhães R., Durães N., Silva M., Silva J., Sencadas V., Botelho G., Gómez Ribelles J.L., Lanceros-Méndez S. (2010). The role of solvent evaporation in the microstructure of electroactive β-poly(vinylidene fluoride) membranes obtained by isothermal crystallization. Soft Mater..

[4] California A., Cardoso V.F., Costa C.M., Sencadas V., Botelho G., Gómez-Ribelles J.L., Lanceros-Mendez S. (2011). Tailoring porous structure of ferroelectric poly(vinylidene fluoride-trifluoroethylene) by controlling solvent/polymer ratio and solvent evaporation rate. Eur. Polym. J..

[5] Lovinger A.J. (1983). Ferroelectric polymers. Science.

[6] Lovinger A.J., Davis D.D., Cais R.E., Kometani J.M. (1987). The role of molecular defects on the structure and phase transitions of poly(vinylidene fluoride). Polymer.

[7] Lloyd D.R., Kim S.S., Kinzer K.E. (1991). Microporous membrane formation via thermally induced phase separation. II. Liquid liquid phase separation. J. Membr. Sci..

[8] Lloyd D.R., Kinzer K.E., Tseng H.S. (1990). Microporous membrane formation via thermally induced phase separation. I. Solid-liquid phase separation. J. Membr. Sci..

[9] Ma W.Z., Chen S.J., Zhang J., Wang X.L. (2010). Kinetics of thermally induced phase separation in the PVDF blend/methyl salicylate system and its effect on membrane structures. J. Macromol. Sci. B Polym. Phys..

[10] Ma W.Z., Chen S.J., Zhang J., Wang X.L., Miao W.H. (2009). Morphology and crystallization behavior of poly(vinylidene fluoride)/poly(methyl methacrylate)/methyl salicylate, and benzophenone systems via thermally induced phase separation. J. Polym. Sci. B Polym. Phys..

[11] Ma W.Z., Chen S.J., Zhang J., Wang X.L., Miao W.H. (2009). Membrane formation of poly(vinylidene fluoride)/poly(methyl methacrylate)/diluents via thermally induced phase separation. J. Appl. Polym. Sci..

[12] Lin Y.K., Tang Y.H., Ma H.Y., Yang J., Tian Y., Ma W.Z., Wang X.L. (2009). Formation of a bicontinuous structure membrane of polyvinylidene fluoride in diphenyl carbonate diluent via thermally induced phase separation. J. Appl. Polym. Sci..

[13] Yang J., Li D.W., Lin Y.K., Wang X.L., Tian F., Wang Z. (2008). Formation of a bicontinuous structure membrane of polyvinylidene fluoride in diphenyl ketone diluent via thermally induced phase separation. J. Appl. Polym. Sci..

[14] Ma W.Z., Wang X.L., Zhang J. (2010). Effect of MMT, SiO_2_, CaCO_3_, and PTFE nanoparticles on the morphology and crystallization of poly(vinylidene fluorid). J. Polym. Sci. B Polym. Phys..

[15] Bosq N., Guigo N., Zhuravlev E., Sbirrazzuoli N. (2013). Nonisothermal crystallization of polytetrafluoroethylene in a wide range of cooling rates. J. Phys. Chem. B.

[16] Gregorio R. (2006). Determination of the alpha, beta, and gamma crystalline phases of poly(vinylidene fluoride) films prepared at different conditions. J. Appl. Polym. Sci..

[17] Kobayashi M., Tashiro K., Tadokoro H. (1975). Molecular vibrations of three crystal forms of poly(vinylidene fluoride). Macromolecules.

[18] Davis G.T., McKinney J.E., Broadhurst M.G., Roth S.C. (1978). Electric-field-induced phase changes in poly(vinylidene fluoride). J. Appl. Phys..

[19] Ma W.Z., Zhang J., Wang X.L. (2008). Formation of poly(vinylidene fluoride) crystalline phases from tetrahydrofuran/*N*,*N*-dimethylformamide mixed solvent. J. Mater. Sci..

[20] Brandrup J., Immergut E.H., Grulke E.A. (1999). Polymer Handbook.

[21] Kim S.S., Lloyd D.R. (1992). Thermodynamics of polymer/diluent systems for thermally induced phase separation: 3. Liquid-liquid phase separation systems. Polymer.

[22] Ma W.Z., Zhang J., Wang X.L. (2007). Effect of initial polymer concentration on the crystallization of poly (vinylidene fluoride)/poly (methyl methacrylate) blend from solution casting. J. Macromol. Sci. B Polym. Phys..

[23] Ma W.Z., Zhang J., van der Bruggen B., Wang X.L. (2013). Formation of an interconnected lamellar structure in PVDF membranes with nanoparticles addition via solid-liquid thermally induced phase separation. J. Appl. Polym. Sci..

